# Bis benzothiophene Schiff bases: synthesis and in silico-guided biological activity studies

**DOI:** 10.3906/kim-2004-78

**Published:** 2020-08-18

**Authors:** Yasemin ÜNVER, Dilek ÜNLÜER, Şahin DİREKEL, Serdar DURDAĞI

**Affiliations:** 1 Department of Chemistry, Faculty of Science, Karadeniz Technical University, Trabzon Turkey; 2 Department of Medical Microbiology, Faculty of Medicine, Giresun University, Giresun Turkey; 3 Computational Biology and Molecular Simulations Laboratory, Department of Biophysics, School of Medicine,Bahçeşehir University, İstanbul Turkey

**Keywords:** Benzo [b] thiophene, Schiff base, binary QSAR models, molecular docking, antimicrobial and antileishmanial activities

## Abstract

Since benzo [ *b* ] thiophene scaffold is one of the privileged structures in drug discovery as this core exhibitsactivities for different biological problems, in this study bis (benzo[ *b* ]thiophene-2-yl) alkyl methanimine derivatives (1-9) were synthesized by reacting benzo[ *b* ]thiophene-2-carbaldehyde with diamines. All newly compounds were characterized by IR, ^1^H NMR and ^13^C NMR spectroscopic methods. Synthesized compounds were investigated using binary QSARbased models on therapeutic activity prediction of synthesized compounds and they showed high predicted activities in following diseases: bacterial, angina, allergy, depression and obesity. Thus, they were then tested for their antimicrobial and antileishmanial activities as a result of this theoretical study. Compound 1(N, N’- (propane-1,3-diyl) bis (1-(benzo [ *b* ] thiophene-2-yl)) methanimine) was found the most active compound in both diseases. Thus, its molecular docking studies were also carried out.

## 1. Introduction

Benzo [b] thiophene derivatives display remarkable biological activities such as antiinflammatory, analgesic, antifungal, antidepressant, antiangiogenic, estrogen receptor modulating, antimitotic, anticancer, kinase inhibitors, antituberculosis, anticonvulsant, antimalarial, anthelmintic, antihyperglycemic and pesticide. Benzothiophene derivatives have been used as potential diagnostic agents and amyloid binding in neurodegenerative diseases, treatment of fatty acid amide hydrolase inhibitors (FAAH), BMP-2 upregulators, Alzheimer’s disease (AD), human nicotinamide phosphoribosyltransferase inhibitors, BRAF kinase inhibitors, Rho kinase inhibitors, selective linear tachykinin NK2 receptor antagonists, protein tyrosine phosphatase 1B inhibitors, histamine H3 antagonists, antiallergic agents and many other activities [1–7]. By replacing the substituents attached to the benzothiophene ring, some of the FDA-approved anticancer drugs such as arzoxifene and raloxifene were also developed [8,9]. Schiff bases, including azomethine functional group (N=CH), possess biological properties such as analgesic, antifungal, antibacterial, antidepressant, anticancer, anticonvulsant and antiinflammatory [10–15]. Schiff bases are also known to be highly effective in synergistic effects on insecticides and plant growth regulators [16–18].

Antibiotics are widely used in the treatment of bacterial infections for many years in the early 20th century. Penicillin was the first antibiotic used to treat bacterial infections. Antibiotics, which are effective by killing or stopping the growth of bacteria, cause the emergence of drug-resistant pathogens and the development of resistance if used unconsciously and excessively. In addition, the long-term use of antibiotics damages our microbiota and losing the beneficial microorganisms has negative consequence. Approximately 16 million people die annually due to bacterial infections, while developing new approaches to combating drug-resistant pathogens and infectious diseases. New substances have been synthesized due to technological developments and have been used in various bacterial infections for therapeutic purposes [19,20].

Leishmaniasis is a disease caused by a protozoon called Leishmania which is transmitted by the bite of infected female sand flies. Poor nutrition lack of sanitation weakened immune system and poverty facilitate spread of the disease in underdeveloped and developing countries. The disease occurs in 3 different forms: cutaneous (most common), visceral (also known as kala-azar and the most serious form of the disease), and mucocutaneous. An estimated 700,000 to 1 million new cases are reported annually, and 26,000 to 65,000 disease-related deaths are reported by the World Health Organization.While cutaneous form is endemic in Southeastern Anatolia, visceral form is mostly seen in Mediterranean, Central Anatolian and Aegean Regions andimportant constitutes an important health problem for Turkey [21,22].

Recent advances made in molecular biology and computational chemistry open new avenues in designing novel therapeutic compounds. Molecular biology has provided the crystal structures and cryo-electron microscopy structures of crucial targets for different diseases, which can be used as accurate templates in modeling studies. Computational chemistry offers a range of simulation, multiscale modeling and virtual screening tools for definition and analysis of protein-ligand, protein-protein interactions. Development of new techniques on statistical methods and free energy simulations help to predict novel optimal ligands. Thus, in this study, we performed in silico-guided biological activity studies for a set of newly synthesized compounds. We aimed to synthesize a new series of Schiff bases including benzo [ *b* ] thiophene for more efficacious biologic activities. In order to predict the biological activities of synthesized compounds before the experimental studies, therapeutic activities of these novel compounds were predicted. The therapeutic activity value (TAV) of each compound was calculated by binary QSAR models of 25 common disease QSAR models. In silico results represented that synthesized compounds may show potential activity against in following diseases: bacterial, angina, allergy, depression and obesity. Therefore, these compounds were then tested for their antimicrobial and antileishmanial activities. Moreover, molecular docking study for one of the most active compounds as antibacterial agent was performed and its molecular interactions were studied.

## 2. Experimental

### 2.1. General method for the synthesis of compounds (1-9)

Benzo [ *b* ] thiophene-2-carbaldehyde (2 mmol) and diamines (1 mmol) were mixed and heated in oil bath without solvent for 2–3 h at 150–160 °C. The reaction content was controlled with TLC examination and cooled to room temperature. The precipitate formed was purified by recrystallization from ethanol-diethylether.

#### 2.1.1. Synthesis of N, N’-(propane-1,3-diyl) bis(1-(benzo[ *b* ] thiophene-2-yl) methanimine) (1):

Yield: 87.00%, m.p. 183–184 °C. IR (KBr, cm^−1^): 3056 (=CH), 2936 (CH), 1633 (C=N), 1600 (C=N), 1527 (C=C); ^1^H NMR (400 MHz, DMSO-d_6_) δ: 8.64 (s, 2H, N=CH), 7.92–7.97 (m, 4H, Arom.H), 7.82 ( bs, 2H,thiop.H), 7.42 (bs, 4H, Arom.H), 3.68 (s, 4H, N-CH_2_) , 1.98 (s, 2H, N-CH_2_-C H_2_); ^13^C NMR (100 Hz, DMSO-d_6_) δ: 156.23, 143.05, 139.99, 139.55, 128.78, 126.64, 125.28, 123.22, 58.28, 32.05.

#### 2.1.2. Synthesis of 1-(benzo[ *b* ]thiophene -2-yl)-N-(4-((benzo[ *b* ]thiophene-2-ylmethylene) amino) butyl) methanimine(2):

Yield: 88.20%, m.p. 166–167 °C. IR (KBr, cm^−1^): 3056 (=CH), 2939 (CH), 1628 (C=N), 1526 (C=N), 1464 (C=C); ^1^H NMR (400 MHz, DMSO-d_6_) δ: 8.61 (s, 2H, N=CH), 7.87–7.97 (m, 4H, Arom.H), 7.79 (2H, bs, thiop.H), 7.37–7.44 (m, 4H, Arom.H), 3.64 (s, 4H, N-CH_2_), 1.68 (s, 4H, N-CH_2_-C H_2_); ^13^C NMR (100 Hz, DMSO-d_6_) δ: 155.87, 143.11, 139.96, 139.65, 128.66, 126.62, 125.22, 123.23, 60.33, 28.64.

#### 2.1.3. Synthesis ofN,N’-(pentane-1,5-diyl)bis(1-(benzo[ *b* ] thiophene-2-yl)methanimine) (3):

Yield: 82.65%, m.p. 110–111 °C. IR (KBr, cm^−1^): 3053 (=CH), 2935 (CH), 1632 (C=N), 1560 (C=N), 1525 (C=C); ^1^H NMR (400 MHz, DMSO-d_6_) δ: 8.58 (s, 2H, N=CH), 7.84–7.93 (m, 4H, Arom.H), 7.75 (2H, bs, thiop.H), 7.37–7.42 (m, 4H, Arom.H), 3.59 (s, 4H, N-CH_2_), 1.65–1.70 (m, 4H, N-CH_2_-C H_2_), 1.35–1.39 (m, 2H, N-CH_2_-CH_2_-C H_2_); ^13^C NMR (100 Hz, DMSO-d_6_) δ :155.73, 143.15, 139.64, 128.56, 126.58, 125.19, 123.20, 60.44, 31.44, 24.87.

#### 2.1.4. Synthesis of 1-(benzo[ *b* ]thiophene-2-yl)-N-(6-((benzo[ *b* ]thiophene-2- ylmethylene) amino) hexyl) methanimine (4):

Yield: 80.28%, m.p. 143–144 °C. IR (KBr, cm^−1^): 3057 (=CH), 2934 (CH), 1628 (C=N), 1560 (C=N), 1526 (C=C); ^1^H NMR (400 MHz, DMSO-d_6_) δ: 8.58 (s, 2H, N=CH), 7.88–7.95 (m, 4H, Arom.H), 7.76 (2H, bs, thiop.H), 7.41 (bs, 4H, Arom.H), 3.58 (s, 4H, N-CH_2_), 1.63 (bs, 4H, N-CH_2_-C H_2_), 1.37 (bs, 4H, N-CH_2_-CH_2_-C H_2_); ^13^C NMR (100 Hz, DMSO-d_6_) δ: 155.73, 143.15, 139.64, 128.56, 126.58, 125.19, 123.20, 60.44, 31.44, 24.87.

#### 2.1.5. Synthesis of N,N’-(heptane-1,7-diyl)bis(1-(benzo[ *b* ] thiophene-2-yl)methanimine) (5):

Yield: 80.85%, m.p. 117–118 °C. IR (KBr, cm^−1^): 3056 (=CH), 2934 (CH), 1673 (C=N), 1629 (C=N), 1517 (C=C); ^1^H NMR (400 MHz, DMSO-d_6_) δ: 8.57 (s, 2H, N=CH), 7.87–7.94 (m, 4H, Arom.H), 7.76 (2H, bs, thiop.H), 7.37–7.43 (m, 4H, Arom.H), 3.56 (s, 4H, N-CH_2_), 1.62 (bs, 4H, N-CH_2_-C H_2_), 1.37 (bs, 6H, N-CH_2_-CH_2_-C H_2_-C H_2_); ^13^C NMR (100 Hz, DMSO-d_6_) δ: 155.63, 143.16, 139.95, 139.64, 128.54, 126.59, 125.19, 123.22, 60.53, 30.70, 28.93, 27.14.

#### 2.1.6. Synthesis of 1-(benzo[ *b* ]thiophene-2-yl)-N-(8-((benzo[ *b* ]thiophene -2- ylmethylene) amino) octyl) methanimine (6):

Yield: 84.85%, m.p. 122–123 °C. IR (KBr, cm^−1^): 3056 (=CH), 2924 (CH), 1628 (C=N), 1525 (C=N), 1465 (C=C); ^1^H NMR (400 MHz, DMSO-d_6_) δ: 8.59 (s, 2H, N=CH), 7.91 (bs, 4H, Arom.H), 7.78 (2H, bs, thiop.H), 7.41 (bs, 4H, Arom.H), 3.39 (s, 4H, N-CH2) , 1.62 (bs, 4H, N-CH_2_-C H_2_), 1.32 (bs, 8H, N-CH_2_-CH_2-_C H_2_-C H_2_); ^13^C NMR (100 Hz, DMSO-d_6_) δ: 155.65, 143.17, 140.91, 128.56, 126.01, 125.20, 123.23, 60.58, 30.78, 29.36, 27.18.

#### 2.1.7. Synthesis of N,N’-(nonane-1,9-diyl)bis(1-(benzo[ *b* ] thiophene-2-yl)methanimine) (7):

Yield: 81.20%, m.p. 115–116 °C. IR (KBr, cm^−1^): 3057 (=CH), 2923 (CH), 1672 (C=N), 1629 (C=N), 1525(C=C); ^1^H NMR (400 MHz, DMSO-d_6_) δ: 8.57 (s, 2H, N=CH), 7.89–7.96 (m, 4H, Arom.H), 7.78 (2H, bs, thiop.H), 7.41 (bs, 4H, Arom.H), 3.56 (s, 4H, N-CH_2_), 1.60 (bs, 4H, N-CH_2_-C H_2_), 1.30 (bs, 10H, N-CH_2_-CH_2_-C H_2−_C H_2_-C H_2_); ^13^C NMR (100 Hz, DMSO-d_6_) δ: 155.64, 143.17, 139.95, 139.66, 128.56, 126.60, 125.21, 123.23, 60.57, 30.77, 29.41, 29.18, 27.18.

#### 2.1.8. Synthesis of 1-(benzo[ *b* ]thiophene-2-yl)-N-(10-((benzo[ *b* ]thiophene-2- ylmethylene) amino) decyl) methanimine (8):

Yield: 83.25%, m.p. 141–142 °C. IR (KBr, cm^−1^): 3050 (=CH), 2924 (CH), 1625 (C=N), 1591 (C=N), 1525(C=C); ^1^H NMR (400 MHz, DMSO-d_6_) δ: 8.58 (s, 2H, N=CH), 7.88–7.94 (m, 4H, Arom.H), 7.77 (2H, bs, thiop.H), 7.40 (bs, 4H, Arom.H), 3.56 (s, 4H, N-CH_2_), 1.58 (bs, 4H, N-CH_2_-C H_2_), 1.28 (bs, 12H, N-CH_2_-CH_2_-C H_2−_C H_2_-C H_2_); ^13^C NMR (100 Hz, DMSO-d_6_) δ: 155.64, 143.18, 139.70, 139.66, 128.55, 126.59, 125.21, 123.23, 60.57, 30.77, 29.83, 29.41, 27.33.

#### 2.1.9. Synthesis of 1-(benzo[ *b* ]thiophene-2-yl)-N-(12-((benzo[ *b* ]thiophene-2- ylmethylene) amino) dodecyl) methanimine (9):

Yield: 80.25%, m.p. 122–123 °C. IR (KBr, cm^−1^): 3054 (=CH), 2918 (CH), 1632 (C=N), 1560 (C=N), 1526 (C=C); ^1^H NMR (400 MHz, DMSO-d_6_) δ: 8.58 (s, 2H, N=CH), 7.90–7.95 (m, 4H, Arom.H), 7.79 (2H, bs, thiop.H), 7.41 (bs, 4H, Arom.H), 3.38 (s, 4H, N-CH_2_), 1.59 (bs, 4H, N-CH_2_-C H_2_), 1.24 (bs, 10H, N-CH_2_-CH_2_-C H_2−_C H_2_-C H_2_), 1.06 (bs, 4H, N-CH_2_-CH_2_-CH_2−_CH_2_-CH_2_-C H_2_); ^13^C NMR (100 Hz, DMSO-d_6_) δ: 155.65, 139.70, 128.57, 126.60, 125.21, 123.23, 60.57, 30.74, 29.40, 29.18.

### 2.2. MetaCore/MetaDrug applications

Studied compounds were examined for their therapeutic activity against 25 common diseases using Meta-Core/MetaDrug platform of Clarivate Analytics (Philadelphia, PA, USA). Training set of compounds were collected from FDA approved compounds, candidate drugs in clinical phases and hit compounds from in vivo activities. Results showed that synthesized compounds can be used as antibacterial agents. Following training and test set compound numbers were used in bacterial QSAR model constructions: Training set N = 530, test set N = 97, sensitivity = 0.87, specificity = 0.90, accuracy = 0.89, Matthews correlation coefficient (MCC) = 0.77.

## 3. Ligand preparation

The synthesized molecules are prepared for further calculations via LigPrep module [23] of Schrodinger’s molecular modeling suite with OPLS2005 force field [24]. Epik [25] was used to determine the protonation states of these compounds at neutral pH.

## 4. Protein preparation

The X-ray diffraction (XRD) structure of the target was used from the Protein Data Bank (PDB) with PDB IDs of 5TW8. Protein preparation module was utilized [26], [27]. Missing side chains are filled via prime module and PROPKA [28]. was subjected to determine the protonation of side chains at physiological pH. Finally, a restrained minimization in OPLS2005 force field was applied to these optimized structures.

## 5. Molecular docking

The molecules which possessed desirable pharmacokinetic and toxicity properties were subjected to a grid-based Glide/XP docking protocol from Schrodinger’s Maestro molecular modeling package [29]. Binding pocket was determined from cocrystallized ligand and default protocol was employed. Shortly, we used flexible docking approach. Hydroxyl and thiol groups of binding pocket residues were allowed for rotation throughout the docking. Extra precision (XP) protocol of Glide was used. Ten poses were requested at the docking and postdocking minimization was performed.

## 6. Results and discussion

### 6.1. Synthesis

Synthesis of bis (benzo[b]thiophene-2-yl) alkyl methanimine derivatives (1-9) was performed according to the reaction outlined in Scheme.

**Scheme:**
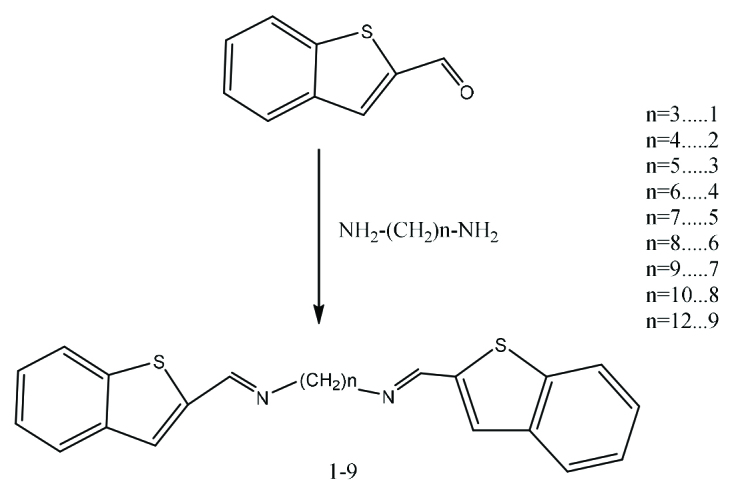
Synthesis of Schiff base derivatives with benzo [b] thiophene (1-9).

The peaks of NH_2_ and C=O belonging to the starting amines and aldehyde respectively disappeared in the IR spectra of the compounds (1-9). Proton signal of imine group (N=CH) obtained by Schiff base reaction was observed as singlet at 8.57–8.64 ppm in the ^1^H-NMR spectra of the compounds (1-9). Aromatic protons belonging to benzo[ *b* ]thiophen ring resonated at 7.37–7.97 ppm and CH_2_ alkyl protons were seen at 1.28–3.68 ppm in the ^1^H-NMR spectra of the compounds as expected from alkyl groups. When carbon spectrawere examined, N=CH imine carbon was observed at 155.64–156.23 ppm in the ^13^C-NMR spectra of compounds 1-9. Aromatic carbon peaks of benzo [ *b* ] thiophene were seen at 123.20–143.19 ppm and alkyl carbon peaks resonated at 24.87–60.58 ppm in the ^13^C-NMR spectra of the compounds. As a result, spectral data supports structures of the compounds (1-9).

### 6.2. In silico predictions of therapeutic activities of synthesized compounds

Binary quantitative structure-activity relationships (QSAR) common disease models from Clarivate Analytics MetaCore/MetaDrug platform were used for the therapeutic activity predictions of synthesized compounds. For this aim, 25 different common diseases binary QSAR models were used. All the nine compounds were screened on MetaCore/MetaDrug platform and therapeutic activity values were predicted. Therapeutic activity values in MetaCore/MetaDrug are normalized between 0 and 1 (while 0 represents inactive compound, 1 represents active compounds). Although predicted therapeutic activity value higher than 0.5 indicates compound that may show activity, in the current study we used a higher cutoff value (0.75) for being in the safe zone. Table S1 at the supplementary information show corresponding predicted therapeutic activity values of each studied compound that has activity value equal or more than 0.75. When we check diseases in Table S1 it can be seen that synthesized compounds can be considered mainly for bacterial, angina, allergy, depression and obesity models. Since the investigated compounds are analogs of each other they showed similar TAV values in certain disease models such as antibacterial profile. Thus, from these diseases we considered to perform antibacterial in vitro assays using these compounds. Furthermore, since known antifungal and antileishmanial effect of benz [ *b* ] thiophene derivatives, we also performed antifungal and antileishmanial in vitro assays for these compounds.

### 6.3. In vitro antileishmanial activity studies

**Figure 1:**
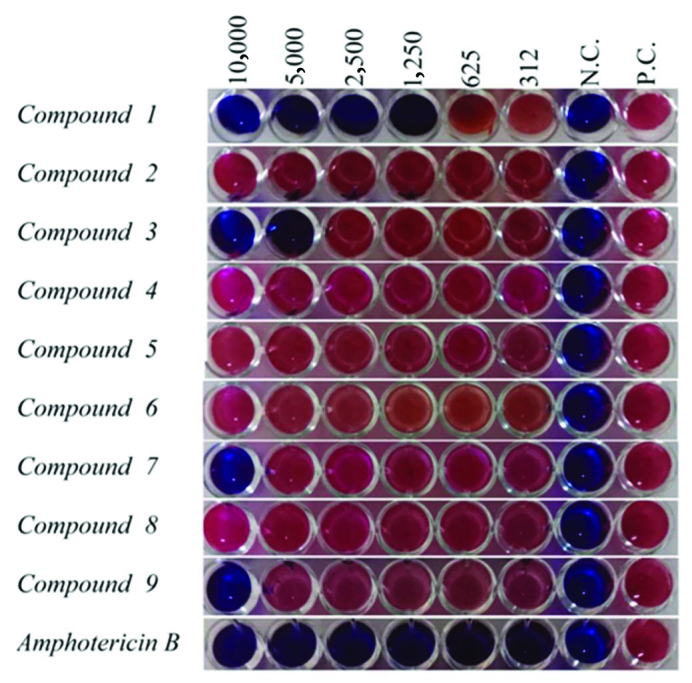
Antileishmanial activity results of the compounds against standard *Leishmania infantum* promastigotes. Control drug: Amphotericin B. Dilution concentrations 10,000 μg/mL to 312 μg/mL. N.C.: Negative control; P.C.: Positive control.

**Figure 2:**
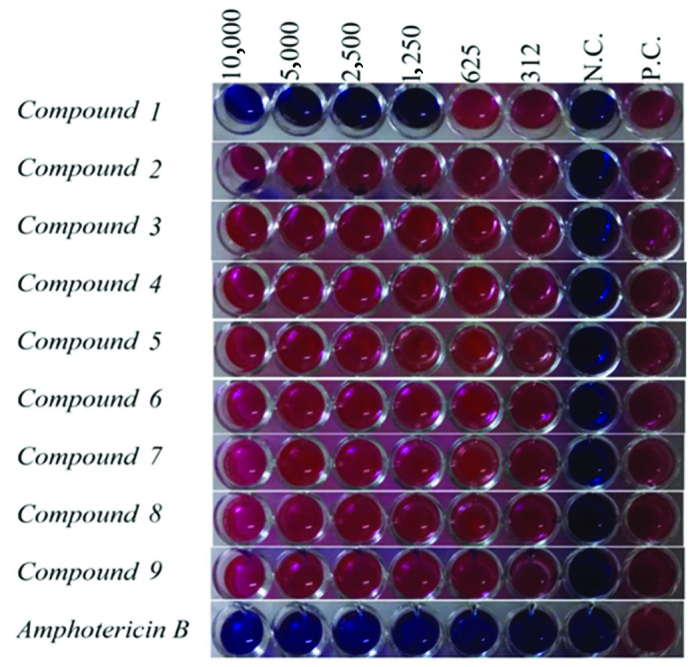
Antileishmanial activity results of compounds against standard *Leishmania tropica* promastigotes. Control drug: Amphotericin B. Dilution concentrations 10,000 μg/mL to 312 μg/mL. N.C.: Negative control; P.C.: Positive control.

Antileishmanial activity results of nine compounds as a result of evaluation Figures 1–2 and minimum inhibitory concentration (MIC) values are given in Table 1. The tests in the positive and negative control wells were found to work as expected.

**Table 1:** Minimum inhibitory concentration (MIC) values of the compounds against *Leishmania infantum* and *Leishmania tropica* promastigotes.

Compounds	*L.infantum* MIC values (μg/mL)	*L.tropica* MIC values (μg/mL)
1	1250	1250
2	>10,000	>10,000
3	5000	>10,000
4	>10,000	>10,000
5	>10,000	>10,000
6	>10,000	>10,000
7	10,000	>10,000
8	>10,000	>10,000
9	10,000	>10,000
Amphotericin B		<312

Compound 1 was found to be the most effective compound (MIC: 1250 μg / mL) among the compounds whose antileishmanial activities were evaluated against *Leishmania infantum* promastigotes. In addition, compounds 3, 7 and 9 were found to have antileishmanial activity at different concentrations (MIC: 5000–10,000 μg/mL).

It was found that other compounds did not have antileishmanial activities against *Leishmania infantum* promastigotes at the studied concentrations. Compound 1 was also found to be the most effective compound against the Leishmania tropica promastigotes from compounds whose antileishmanial activities were evaluated (MIC: 1250 μg/mL). It was determined that other compounds did not have antileishmanial activities against *Leishmania tropica* promastigotes at studied concentrations.

The study of Maina et al. revealed the antileishmanial activity of *Clerodendrum myricoides* and *Salvadora persica* . They reported, *Clerodendrum myricoides* water extract demonstrated the best potential antileishmanial activity against *Leishmania major* promastigotes (MIC = 625 μg/mL). Also, the dichloromethane and petroleum ether extract were reported moderate to weak activity against *Leishmania major* promastigotes (MIC = 1250 μg/mL; 2500 μg/mL) and amastigotes respectively [30].

In the study of Ogeto et al., *Aloe secundiflora* water extract was found active against L. major at the lowest concentration of 2000 μg/mL. Methanollic plant extract reported less antileishmanial activity as compared to Pentostam and amphotericn B with MIC of 1000 μg/mL, 250 μg/mL and 125 μg/mL, respectively. They marked higher antileishmanial activities though not of comparative concentrations than most of the reference drugs. The results also showed that plant extracts had lower toxicity against Vero cells as compared to the standard drug amphotericin B [31].

Amphotericin B, which is used as a standard drug, was found to be effective at all concentrations (MIC: <312 μg / mL) for both leishmania species.

### 6.4. In vitro antibacterial and antifungal activity studies

**Figure 3:**
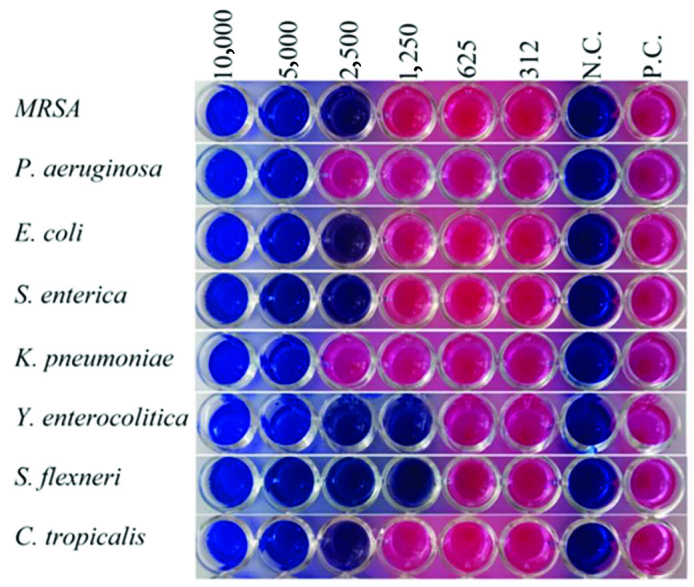
Antibacterial and antifungal activity results of compound 1. Dilution concentrations: 10,000 μg/mL–312 μg/mL. N.C.: Negative control; P.C.: Positive control.

Different concentrations of some compounds against standard bacterial isolates and yeast isolates were found to have antimicrobial activity, while some compounds were found to be ineffective at the concentrations studied. The test images of compound 1 which have the most effective antibacterial and antifungal effect are given in the Figure 3. The MIC values of the compounds are given in Table 2. It was determined that the compounds showed antibacterial and antifungal activity against 6 of the studied standard bacteria and yeast isolates.

**Table 2:** Minimum inhibitory concentration (MIC) values of all compounds against bacteria and fungi.

								
1	2500	2500	5000	1250	2500	1250	5000	2500
2	>10000	>10000	>10000	10000	>10000	10000	10000	>10000
3	>10000	>10000	>10000	10000	>10000	>10000	10000	>10000
4	>10000	>10000	>10000	10000	>10000	10000	10000	>10000
5	>10000	>10000	>10000	10000	>10000	>10000	10000	>10000
6	>10000	>10000	>10000	10000	>10000	10000	10000	>10000
7	>10000	>10000	>10000	10000	>10000	>10000	10000	>10000
8	>10000	>10000	>10000	10000	>10000	10000	10000	>10000
9	>10000	>10000	>10000	10000	>10000	10000	10000	>10000

The compound 1 showed the strongest antibacterial effect against *Shigella flexneri* and *Yersinia enterocolitica* (MIC:1250 μg/mL) (Figure 3). Compound 1 was found to be effective against all bacteria and yeast (Table 2). Antibacterial and antifungal activity were determined in 6 compounds (1, 2, 4, 6, 8 and 9).

When the results of synthesized compounds were evaluated, it was found that compound 1 was the most effective compound for all bacteria and yeast isolates studied. Compound 1 was found to have different levels of antimicrobial activity against *Shigella flexneri* , *Yersinia enterocolitica* , *Staphylococcus aureus* (MRSA), *Escherichia coli* , *Salmonella enteritidis* , *Candida tropicalis* , *Pseudomonas aeruginosa* , and *Klebsiella pneumoni* (respectively MIC values: 1250 μg/mL, 1250 μg/mL, 2500 μg/mL, 2500 μg/mL, 2500 μg/mL, 2500 μg/mL,5000 μg/mL, and 5000 μg/mL).

It is found that *Klebsiella pneumoniae* is the least affected one by the compounds. All concentrations of the compounds 3, 5 and 7 were found to have no effect on any bacterial and yeast isolates (MIC: >10,000 μg/mL).

Thus, because of its promising results at both disease models, compound 1 can be considered as lead compound. However, further animal in vivo studies must be carried out.

**Figure 4:**
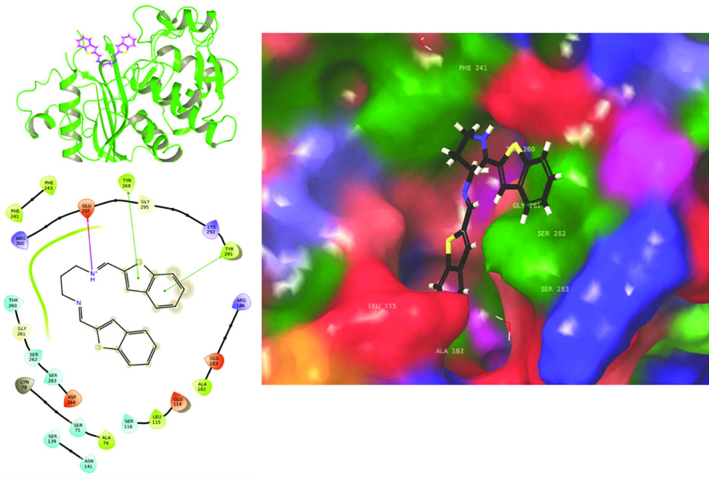
2D and 3D ligand interactions diagrams of compound 1 at the binding pocket of 5TW8 PDB coded structure.

Since the most active compound was found as compound 1 as antibacterial agent, we used crystal structure of wild-type *S. aureus* penicillin binding protein 4 (PBP4), PDB ID: 5TW8, and performed dockin simulations. Figure 4 shows 2D and 3D ligand interactions diagram of compound 1 at the binding pocket of the target structure. It can be seen that main nonbonded interactions are constructed with Tyr291 and Tyr268 (π-π stacking interactions) and with Glu297 (ionic interactions). These strong interactions (docking score, -8.33 kcal/mol) show that compound 1 can be considered as promising anti-bacterial agent. Both in silico predictions (ligand-based and target-driven based) as well as performed in vitro assays validate each other for the antibacterial effect of the novel compound (compound 1). Our hybrid molecular modeling approache (combined ligand-based and structure-based) predicted the biological activity of a set of new compounds and these predictions were investigated by in vitro studies.

## 7. Conclusion

Nine bis (benzo[b]thiophen-2-yl) alkyl methanimine derivatives were synthesized. All newly compounds were characterized by IR, ^1^H NMR and ^13^C NMR spectroscopic methods.Synthesized compounds were investigated using 25 different binary QSAR disease models. Results showed that synthesized compounds may be considered mainly for bacterial, angina, allergy, depression and obesity models. Our in silico-guided design study lead to a hit compound (Compound 1) for its antibacterial effects. In vitro antileishmanial, antifungal and antibacterial activity studies were performed on all synthesized compounds. According to the test results; Compound 1 showed antileishmanial activity (MIC = 1250 μg/mL) and it was concluded that further studies could be continued for this compound 1. The purpose of the control drug study was made to test whether the experimental study was working properly. The substances studied showed antileishmanial activity even if they had a higher value than the MIC of the standard drug Amphotericin B. In this antileishmanial activity study, while aiming to determine the concentration of the compound that is effective against leishmania, the reliability, toxic effect and side effects of the substances should also be evaluated for new drug candidates. Due to the toxic effects of currently used drugs and the increasing resistance to these drugs, the discovery and development of new therapeutic agents is important.Compound 1 also showed better antibacterial and antifungal activity.

Supplementary Materials
