# Supplementary material for: Bis benzothiophene Schiff bases: synthesis and in silico-guided biological activity studies
Source: Turk J Chem. 2020 Aug 18;44(4):1164–76. doi: 10.3906/kim-2004-78 (PMC7751927; doi:10.3906/kim-2004-78)
Supplement: Supplementary file 1 — Supplementary Materials [file turkjchem-44-1164-sup001.pdf]

Table S1. Therapeutic activity value (TAV) predictions using binary QSAR models.

Compound 1

Disease	TAV
Bacterial	0.78
Angina	0.77
Depression	0.75
Allergy	0.75

Compound 2

Disease	TAV
Bacterial	0.81
Angina	0.79
Allergy	0.77

Compound 3

Disease	TAV
Bacterial	0.81
Angina	0.79
Allergy	0.77

Compound 4

Disease	TAV
Bacterial	0.82
Angina	0.79
Allergy	0.77
Obesity	0.75

Compound 5

Disease	TAV
Allergy	0.77
Angina	0.77
Obesity	0.75

Compound 6

Disease	TAV
Allergy	0.77
Angina	0.77
Depression	0.75
Obesity	0.75

Compound 7

Disease	TAV
Allergy	0.77
Angina	0.77
Obesity	0.75

Compound 8

Disease	TAV
Bacterial	0.82
Allergy	0.77
Alzheimer	0.76
Obesity	0.75

Compound 9

Disease	TAC
Bacterial	0.82
Allergy	0.77
Angina	0.76
Obesity	0.75

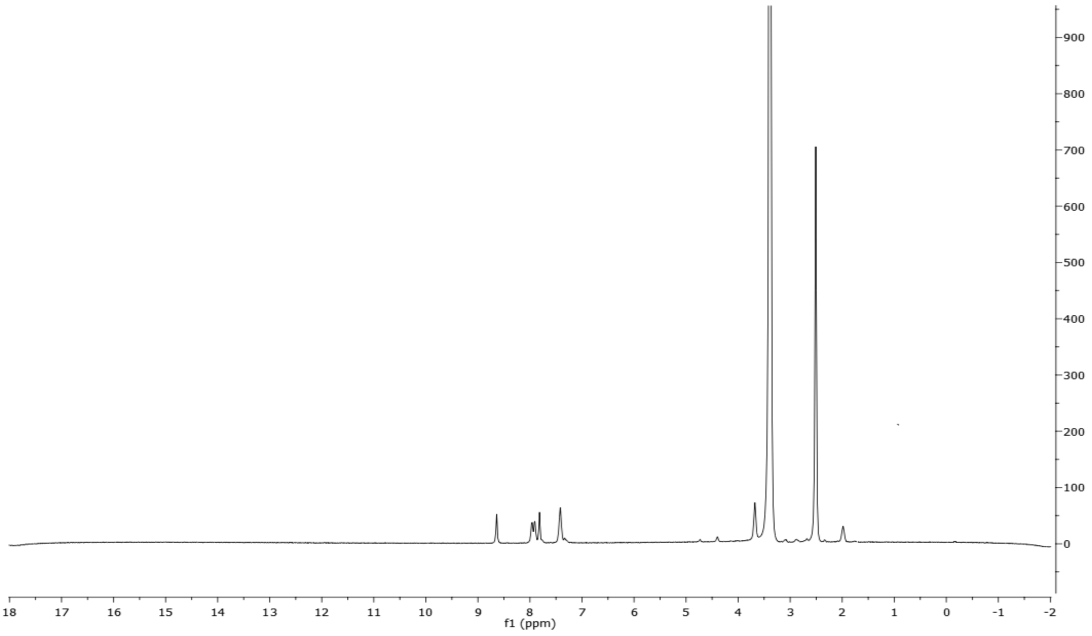

^{1}H-NMR spectrum of compound 1.

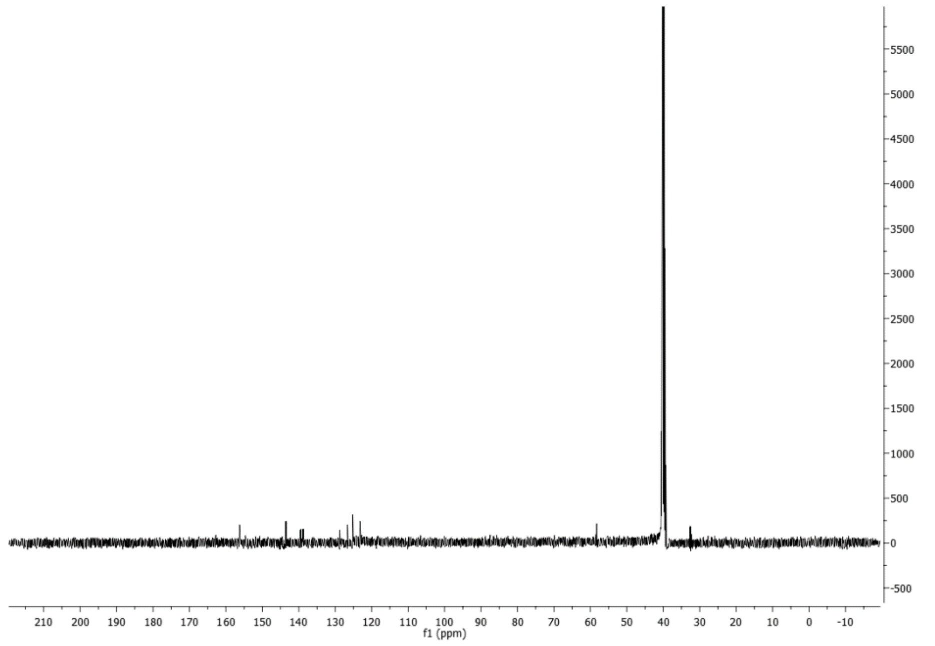

^{13}C-NMR spectrum of compound 1.

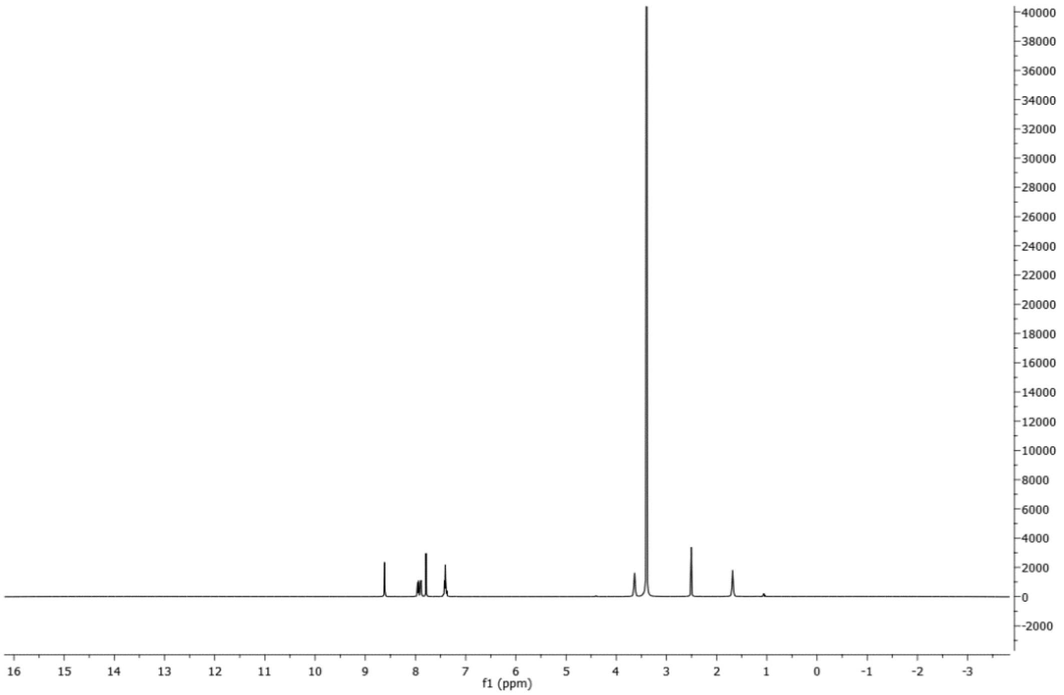

^{1}H-NMR spectrum of compound 2.

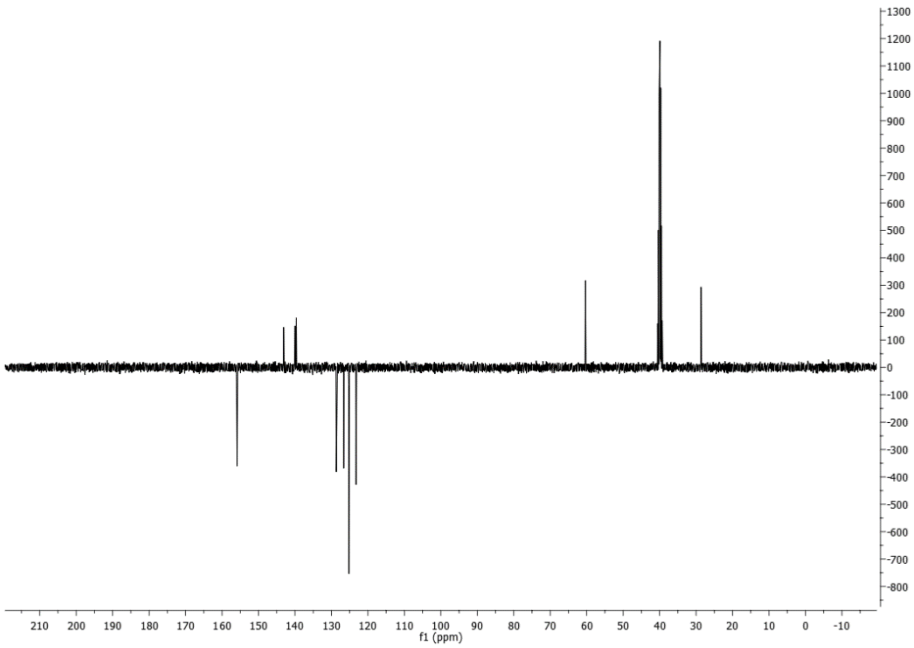

^{13}C-NMR spectrum of compound 2.

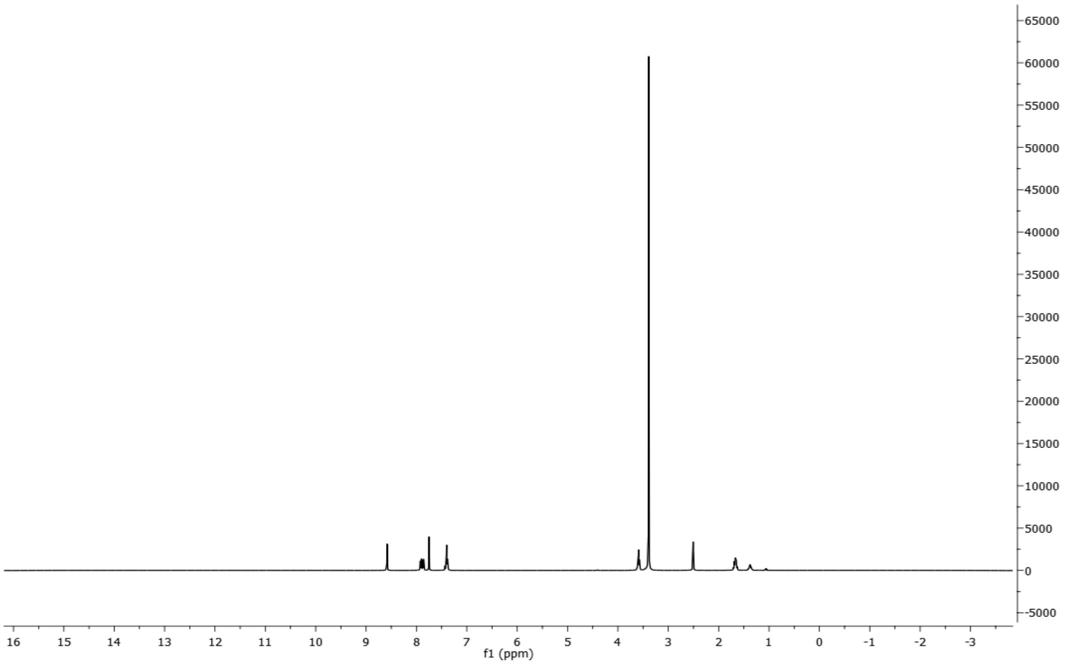

^{1}H-NMR spectrum of compound 3

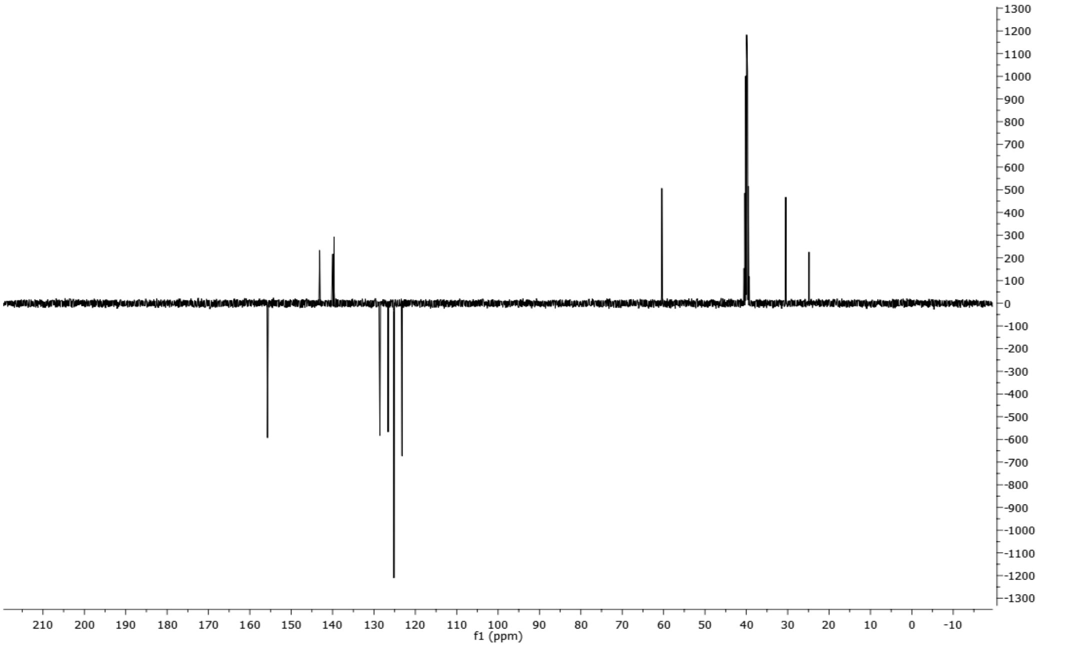

^{13}C-NMR spectrum of compound 3.

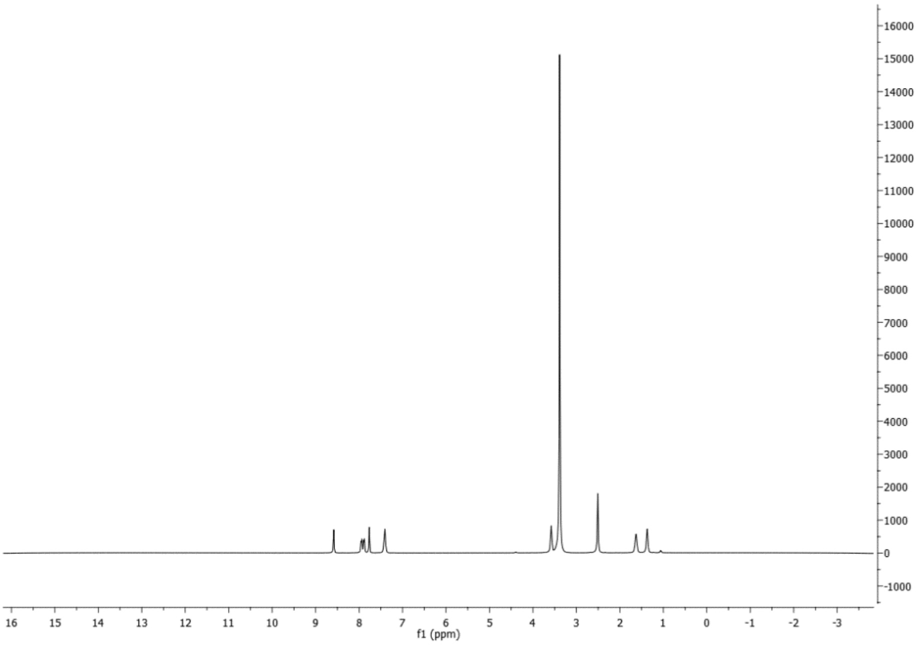

^{1}H-NMR spectrum of compound 4.

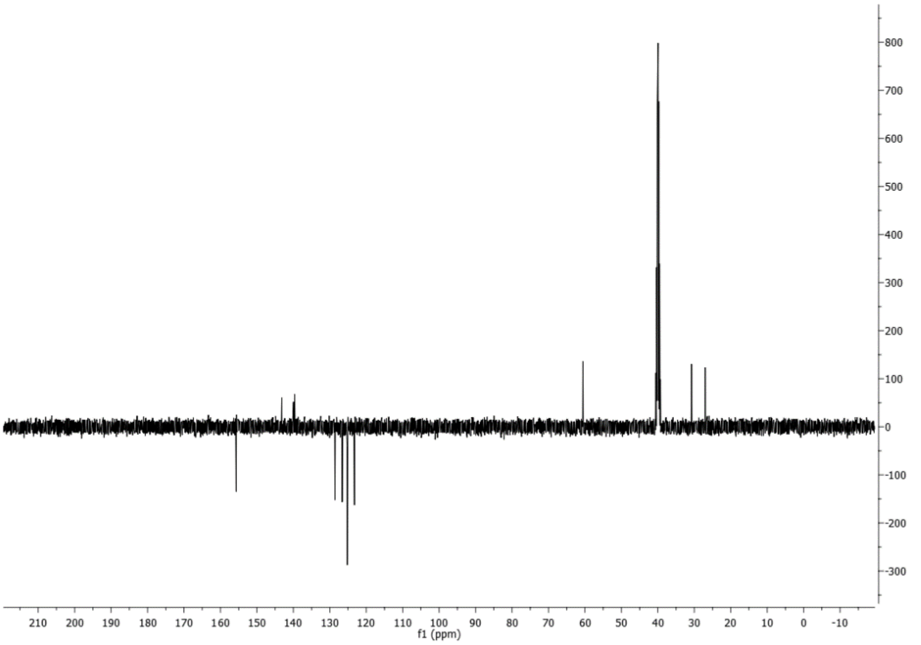

^{13}C-NMR spectrum of compound 4.

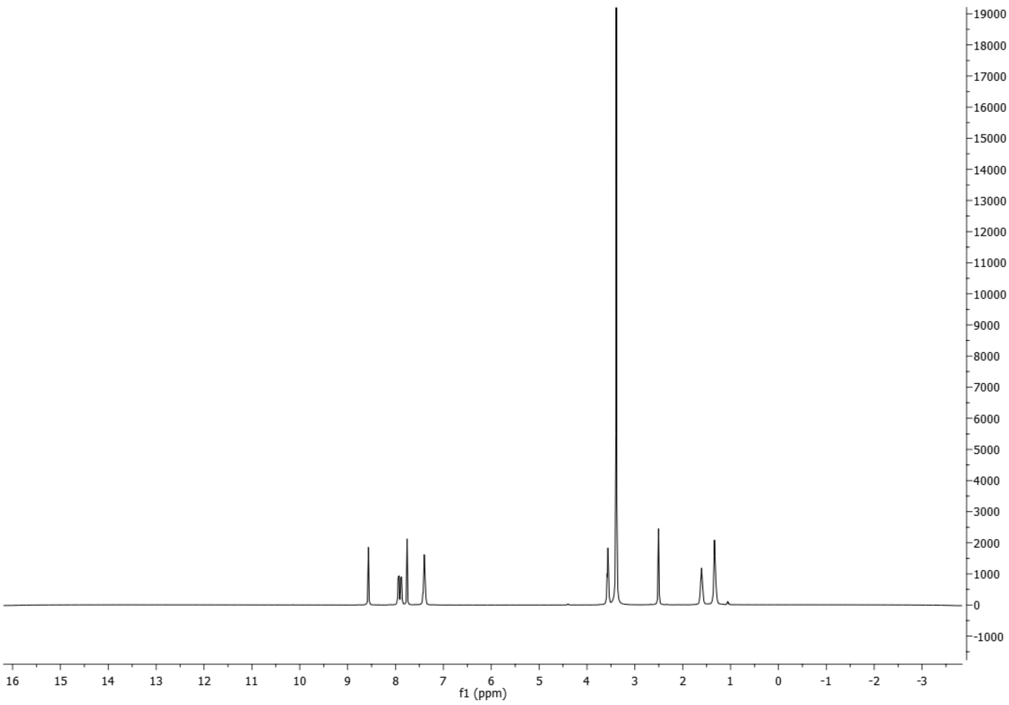

^{1}H-NMR spectrum of compound 5.

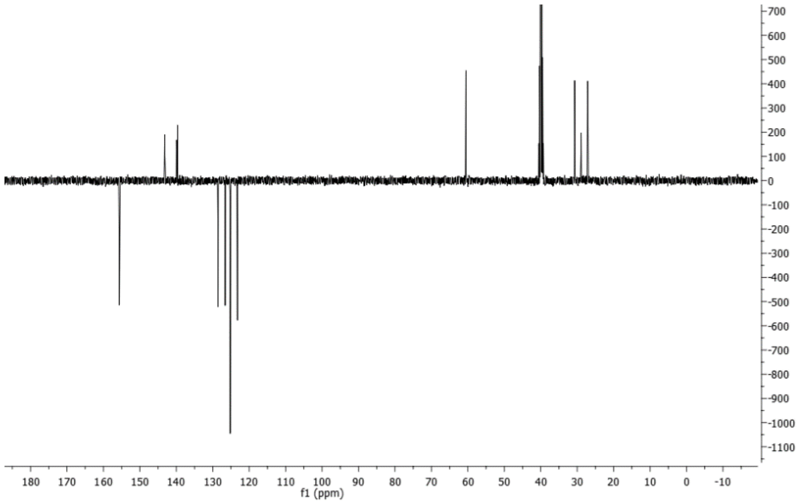

^{13}C-NMR spectrum of compound 5.

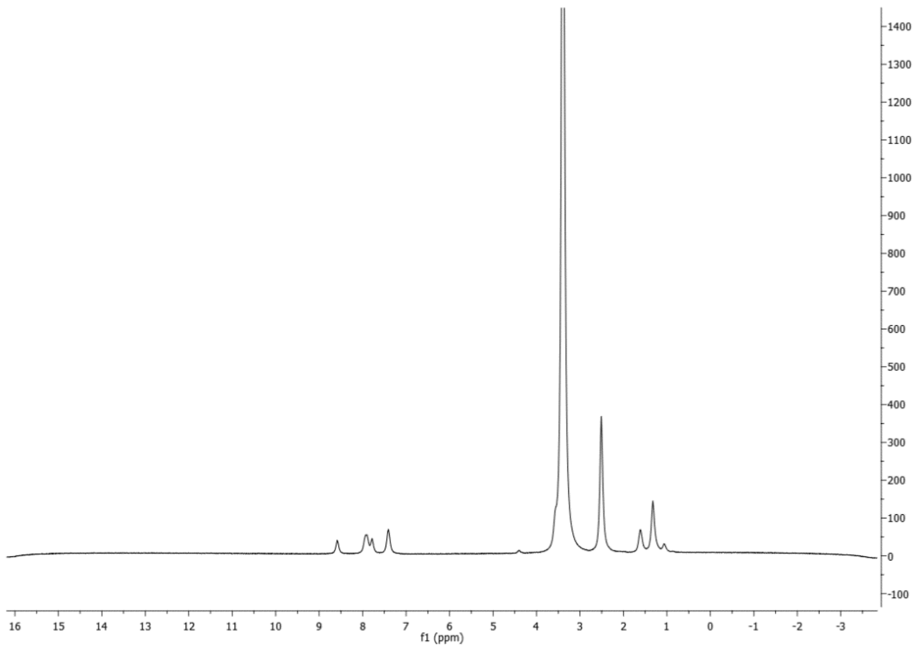

^{1}H-NMR spectrum of compound 6.

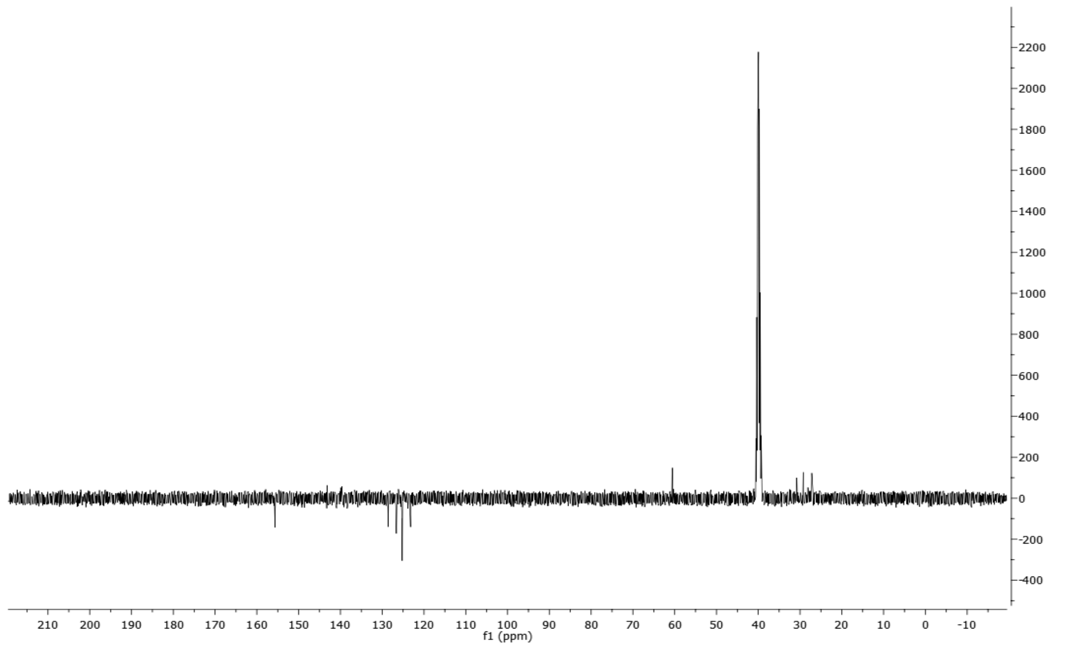

^{13}C-NMR spectrum of compound 6.

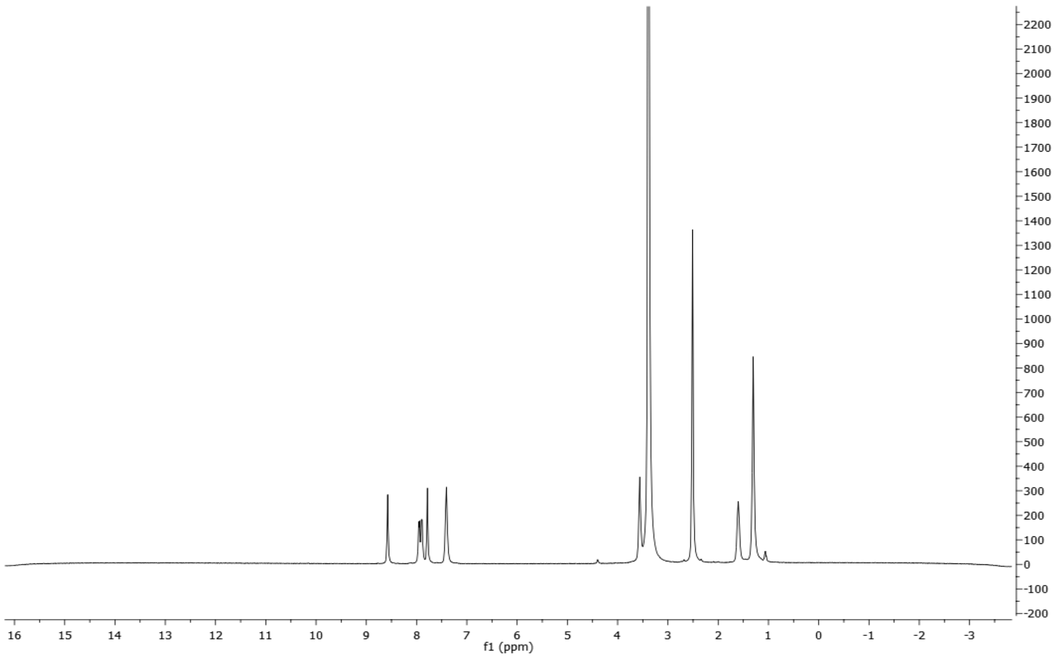

^{1}H-NMR spectrum of compound 7.

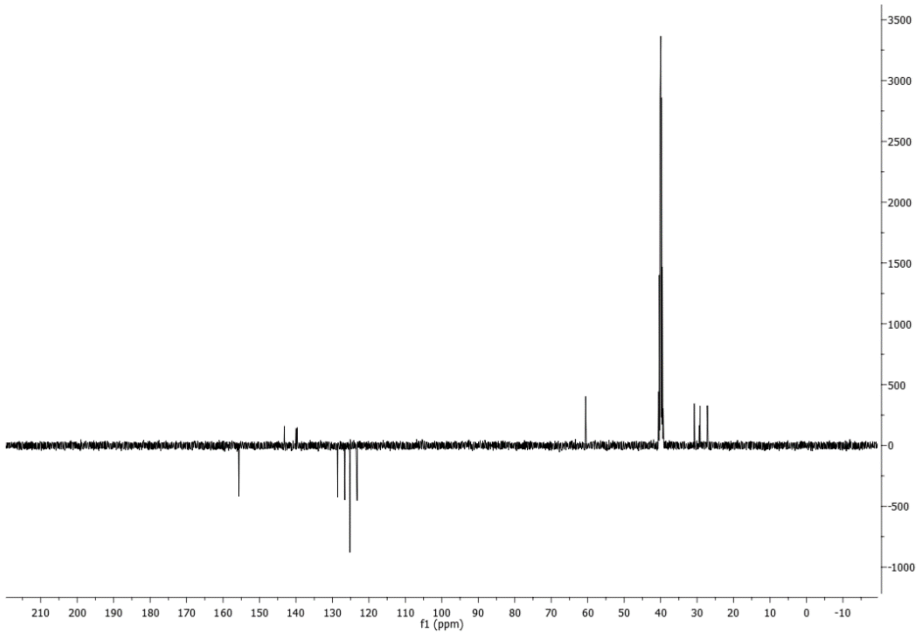

^{13}C-NMR spectrum of compound 7.

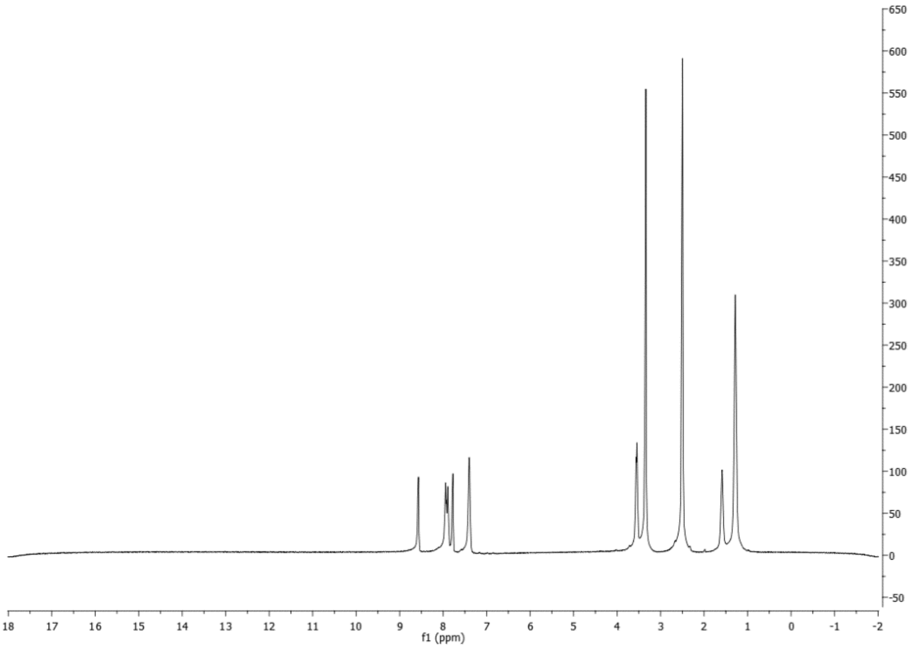

^{1}H-NMR spectrum of compound 8.

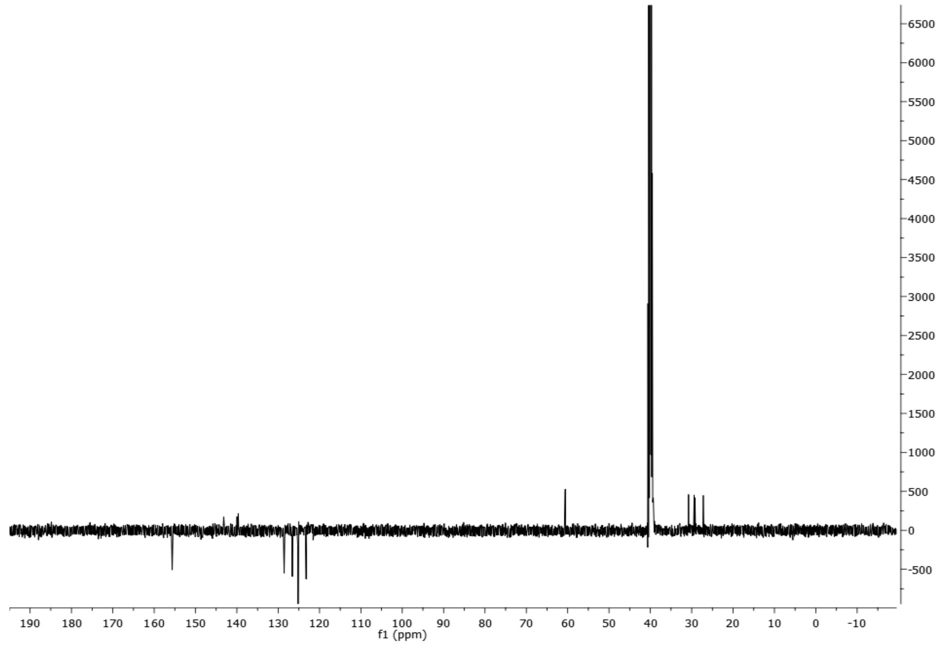

^{13}C-NMR spectrum of compound 8.

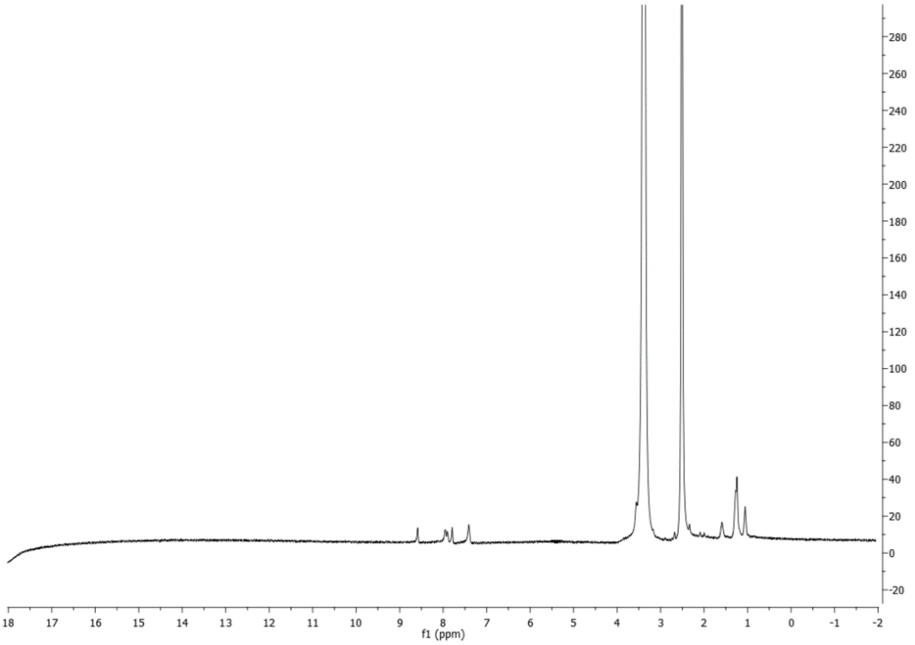

^{1}H-NMR spectrum of compound 9.

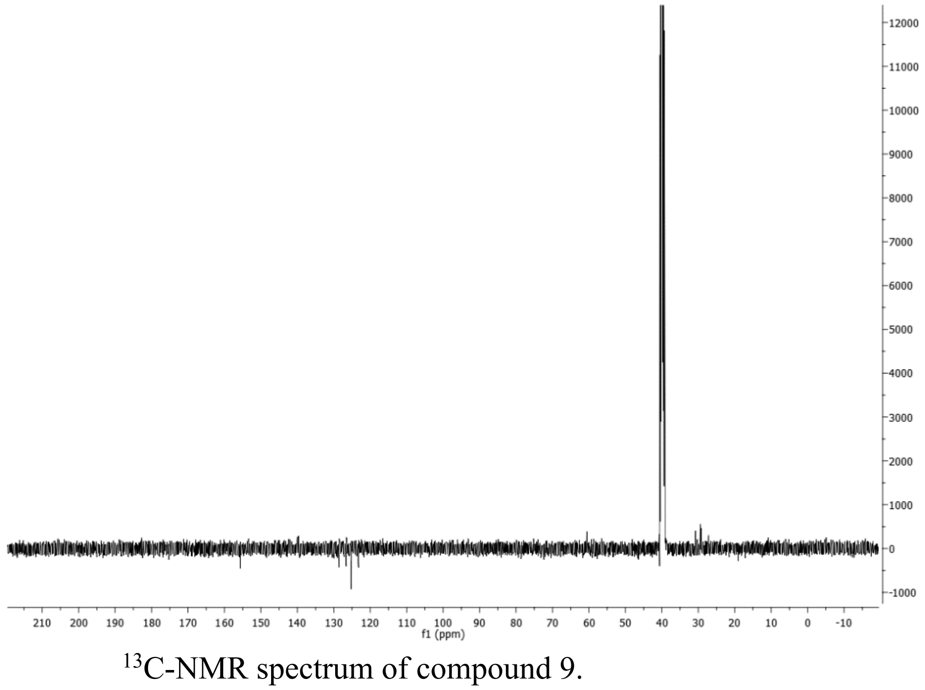

^{13}C-NMR spectrum of compound 9.
